# Online peer support training to promote adolescents’ emotional support skills, mental health and agency during COVID-19: Randomised controlled trial and qualitative evaluation

**DOI:** 10.1007/s00787-021-01933-0

**Published:** 2022-02-17

**Authors:** Gabriela Pavarini, Tessa Reardon, Anja Hollowell, Vanessa Bennett, Emma Lawrance, Peer Support Young People’s Advisory Group, Ellie Brooks-Hall, Ashley Foster-Estwick, Damian Omari Juma, Peter Lewis, Lucy Power, Maia Rogers, Vanessa Pinfold, Ilina Singh

**Affiliations:** 1 Department of Psychiatry and Wellcome Centre for Ethics and Humanities, University of Oxford, Oxford, UK; 2 Department of Experimental Psychology and Department of Psychiatry, University of Oxford, Oxford, UK; 3 The McPin Foundation, London, UK; 4 Imperial College London and Mental Health Innovations, London, UK

**Keywords:** Peer support, COVID-19, Empowerment, Mental health, Adolescents, Social skills

## Abstract

Adolescents often look to their peers for emotional support, so it is critical that they are prepared to take on a supportive role, especially during a health crisis. Using a randomised controlled trial (ISRCTN99248812, 28/05/2020), we tested the short-term efficacy of an online training programme to equip young people with skills to support to their peers’ mental wellbeing during the COVID-19 pandemic. In June 2020, one-hundred UK adolescents (aged 16–18) recruited through social media were randomly allocated (1:1) to immediate 5-day peer support training or a wait-list, via an independently generated allocation sequence. Primary outcomes were indicators of ability to help others (motivation, perceived skills, frequency of help provided, compassion to others and connectedness to peers). Secondary outcomes included emotional symptoms, mental wellbeing, and indicators of agency (civic engagement and self-efficacy). We also collected qualitative reports of participants’ experience. Assessments were completed at baseline and 1 week post randomisation (primary endpoint), and up to 4 weeks post randomisation (training group only). The training increased support-giving skills, frequency of providing support, compassion and peer connectedness (medium–large-effect sizes), but not motivation to provide support, 1 week post randomisation, compared to controls. Gains in the training group were maintained 4 weeks post randomisation. Training also improved adolescents’ mental health and agency, and qualitative reports revealed further positive outcomes including increased self-care and empowerment. Leveraging digital platforms that are familiar to young people, peer support training has the potential to enable adolescents to support their own and their peers’ mental wellbeing during a health crisis.

**Supplementary Information:**

The online version contains supplementary material available at 10.1007/s00787-021-01933-0.

## Introduction

The COVID-19 outbreak has had a major psychosocial impact on the lives of young people [1, 2]. The challenges posed by the pandemic have given rise to feelings of lack of control, loneliness and anxiety, and significant increases in mental ill-health [3, 4], posing an urgent need for targeted interventions. To ensure their specific needs are addressed, it is critical that young people are involved as active stakeholders in priority setting, as well as in the design, testing and delivery of interventions, rather than as passive ‘recipients’ of support [5, 6].

In March 2020, as the pandemic accelerated, we conducted patient and public involvement (PPI) consultations with adolescents aged 14–25 from two UK-based Young People’s Advisory Groups (YPAGs) to help set priorities for intervention research. The young people consulted expressed a clear desire to be active stakeholders in easing the mental health burdens of the pandemic. While many aspired to provide emotional support to their friends and peers during this time, they felt that they lacked the skills to do so. In direct response to these expressed needs, we focused this project on peer support, aiming to equip young people with the skills to reach out to their peers and support their mental health and wellbeing.

Peer support is defined as the process of help-giving and help-receiving between individuals who share characteristics or lived experiences [7]. In the context of mental health, it has been framed as an approach that foregrounds values-led relationships [8, 9]. Evidence indicates that having positive peer relationships in adolescence predicts greater wellbeing [10]. However, evidence from intervention studies is mixed: while some studies suggest positive outcomes for supported peers, others indicate null results (see [11, 12] for systematic reviews of online and school interventions, respectively). This inconsistency may be partly due to the training young people receive to carry out the role [13].

Peer support training programmes are rarely examined in isolation. Existing studies, exclusively school-based, suggest that training brings marginal improvements to peer supporters’ self-confidence [12]. However, to our knowledge, only one study evaluated outcomes directly related to adolescents’ ability to help others. The results showed social connectedness and support-giving increased among adolescent peer supporters who received training, compared to controls [14]. Peer support training also has the potential to heighten young people’s ‘agency capabilities’ (i.e. their ability to pursue valued outcomes, such as making a difference to the community) [15] and bring benefits for their own mental wellbeing, but these outcomes remain largely unexplored. To develop effective peer support interventions, it is first critical to develop and evaluate training to prepare young people for the role. Moreover, COVID-19 restrictions and disruptions have magnified the need for virtual training models [16].

### Goals and aims

Our study addresses the need for peer support training programmes targeted to the COVID-19 pandemic, and the lack of evidenced outcomes of peer supporter training. The project focused on young people aged 16–18 years, an age group experiencing significant disruption to their education or early working life during the COVID-19 pandemic [17]. We partnered with Youth Era, a charity specialising in online peer support for youth (www.youthera.org), and YPAG set up for this project, to design an online training course to equip young people with skills to support the mental health and wellbeing of their friends and peers. The course equipped young people to provide peer support to their communities and social networks in naturally occurring instances, outside of professional services.

Through a randomised controlled trial, we investigated whether being trained as a peer supporter promotes:

i. young people’s ability to support others during the COVID-19 pandemic, including motivation to provide peer support, perceived support-giving skills, frequency of support provided, compassion to others, and connectedness to peers,
ii. their own mental wellbeing and reduces emotional symptoms, and
iii. their sense of agency, including self-efficacy and civic engagement

We investigated short-term benefits, relative to a wait-list control, and explored young people’s experiences of the online training and self-reported impacts.

## Methods

### Co-producing research with young people

A YPAG was formed specifically for this project, comprising six young people aged 16–28 (2 males, 4 females). Young people were recruited from three existing advisory groups that contributed to our initial PPI work: The McPin Young People’s Network (https://mcpin.org/young-peoples-network/), the NeurOX YPAG (https://oxneurosec.com/involvement/young-peoples-advisory-group-ypag/) and the Lancet Young Leaders for Global Mental Health (https://globalmentalhealthcommission.org/youth-campaign/). The YPAG informed all aspects of the study, from design to dissemination (Table S1). The McPin Foundation led the youth involvement, with support from the Oxford Neuroscience, Ethics and Society Group.

### Recruitment

The study protocol was approved by the University of Oxford Interdivisional Medical Sciences Ethics Committee (R69810/RE001). Participants were recruited through posters and advertisements on social media, from May 23 to May 28, 2020. To help overcome potential sampling bias, we used three different adverts on social media, including an illustration and two posters (one depicting a female and another a male model); adverts were set up to target adolescents aged 16–18 years, anywhere in the UK. To be eligible, participants needed: to be aged 16–18, UK resident, have sufficient English and ability to complete training and measures independently, consent to randomisation, and access to Wi-Fi, computer, camera and speakers. Interested participants completed an online “Expression of Interest Form”. Those identified as potentially eligible were invited for a short call with the training instructors to confirm eligibility and suitability for the course. Once confirmed, written informed consent was obtained and participants completed baseline measures. Recruitment ceased once the target sample size was achieved.

### Randomisation and masking

After baseline measures were completed, participants were randomly assigned to immediate training or wait-list control in a 1:1 ratio. The random allocation sequence was computer-generated by an independent researcher who had no contact with research participants. Post-randomisation questionnaires were circulated via email by a researcher blind to allocation. Participants completed all assessments independently online.

### Procedures

Participants randomised to the training arm completed the peer support training course between the 8 and 12 June 2020. Both arms were reassessed 1 week post randomisation, and wait-list participants were then offered the peer support training (22–26th June). Three weekly follow-up assessments of the primary outcomes were circulated to the training arm only. All participants received a certificate of course completion and a £15 voucher for completing study measures.

### Peer support training

The training course, titled Uplift Peer Support Training, was designed by Youth Era with input from the YPAG and researchers. The course was delivered via Zoom by a team of specialist Youth Era peer supporters over 5 consecutive days (4 h/day). Interactive and informative sessions were delivered by peer support experts to the full group of 50 youth, and sharing and hands-on activities were mostly delivered in small groups of 7 via breakout rooms or WhatsApp, each led by a group facilitator. Participants were asked to keep their cameras on during the Zoom sessions, and encouraged to participate actively in the WhatsApp discussions. The course included modules on establishing rapport, active listening, grief and trauma, confidentiality, self-care, coping strategies, crisis management, signposting and referrals, and making a difference to the community. Examples and activities reflected challenges of the pandemic from the perspective of UK youth. Further information about training content is available from authors on request.

### Quantitative outcomes

Assessment points were baseline (before randomisation), 1-week post randomisation (primary endpoint), and 2, 3 and 4 weeks post randomisation (training arm only). Measures assessing all outcomes were administered at baseline and the primary endpoint in both arms; training arm follow-ups included measures of primary outcomes. To characterise the sample, participants reported their age, gender and ethnicity, and completed the Family Affluence Scale [18], an adolescent self-report measure of socioeconomic status.

#### Primary outcomes

Our primary outcomes were the following indicators of the ability to provide support to peers during the COVID-19 pandemic among both arms 1 week post randomisation: (i) motivation to provide support; (ii) perceived support-giving skills; (iii) frequency of support provided; (iv) compassion towards others; and (v) connectedness to peers.

Motivation to provide support, perceived support-giving skills and frequency of support provided were each established using corresponding items from the Adolescent Social Connection Coping During COVID Scale [19] (motivation: 4 items; total score: 4–24; skills: 4 items, total score: 4–28; frequency: 4 items, total score: 4–28). Compassion towards others was assessed using the Compassion to others-Engagement subscale (6 items; total score: 6–60) and the Compassion to others-Action subscale (4 items; total score: 4–40) [20]. Minor amendments were made to the wording of the Action subscale items to assess perceived ability to take actions, rather than to actual behaviours which participants were unlikely to have had the opportunity to engage in within the short-assessment period (Table S2). Connectedness to peers was determined using the Inclusion of Other in the Self [21] single item pictorial measure of closeness (scored 1–7).

#### Secondary outcomes

Secondary outcomes included mental wellbeing and emotional symptoms, and indicators of agency (self-efficacy and civic engagement) among both arms 1 week post randomisation. The Warwick–Edinburgh Mental Wellbeing Scale [22] (WEMWS; 14 items; total score: 14–70), and the Strengths and Difficulties Questionnaire-Emotional symptoms subscale [23] (SDQ-E; 5 items, total score: 0–10) were used to assess mental wellbeing and emotional symptoms, respectively. Self-efficacy was established with the General Self-Efficacy Scale [24] (GESS; 10 items, total score: 10–40) and civic engagement was assessed using the Attitudes and Behaviour subscales of the Civic Engagement Scale [25] (CES-Attitudes; 8 items, total score: 8–56; CES-Behaviour; 6 items; total score 6–42). We made minor amendments to the wording of the CES-Behaviour items to assess perceived ability to engage in behaviours, rather than actual behaviours (Table S2). Further, secondary outcomes assessed in the training arm only included indicators of the ability to provide support (motivation, skills, frequency and connectedness to peers) and who participants reported helping (e.g., friends), up to 4 weeks post randomisation.

### Qualitative outcomes

Open-ended questions assessed participants’ perceived impact of training, use of peer support skills and intentions to use peer skills in the future in the training arm. Participants were asked (i) whether the training impacted their life in any way, and prompted to describe positive and negative impacts (1 week post randomisation); (ii) to provide an example of how they used their “peer support skills” over the past week (2, 3 and 4 weeks post randomisation); and (iii) if and how they planned to use what they learnt from the training in their “life moving forward” (1 week and 4 weeks post randomisation).

### Statistical analysis

#### Sample size

The target sample size was determined to provide 80% power to detect a meaningful difference between the two groups on primary and secondary outcomes. We assumed that we would retain 90% of participants to 1 week post randomisation, and the sample of 100 provided 80% power and (2 sided) 5% significance levels to detect differences between training and control groups of *F* = 0.3 (medium-effect size).

#### Analysis

An analysis plan detailing all pre-specified statistical analyses was published (http://www.isrctn.com/ISRCTN99248812), before analyses were conducted. Baseline characteristics were summarised for each group and the total sample using descriptive statistics. A series of analysis of covariance (ANCOVAs) were used to compare training and control groups on each primary and secondary outcome 1 week post randomisation, adjusting for corresponding baseline score, gender and age. Partial eta-squared was used to measure the effect size for each outcome. To assess the robustness of the results, ANCOVAs were repeated using pre–post change scores and participants aged 16 only. The results of these sensitivity analyses were similar to the main analyses and did not alter the conclusions so are not reported here.

To examine indicators of the ability to provide support up to four weeks post randomisation for the training group, we used a series of repeated measures analysis of variance. We used the last observation carried forward approach to manage missing follow-up data. To assess the impact of these missing data, we repeated these analyses using participants with complete data only (n = 39). The results of these complete data analyses were similar to the main analyses and did not alter the conclusions, so are not reported here.

To maintain an overall type 1 family error rate of 0.05 for the primary outcomes, a Bonferroni adjustment method was used and a *p* value < 0.0083 (0.05/6) was considered statistically significant for each primary outcome. Secondary outcomes were treated as exploratory and so no adjustment for multiple testing was made [26, 27] and p < 0.05 was considered statistically significant for each secondary outcome.

Statistical analyses were done with SPSS version 26. The trial was registered on the ISCRN registry, number ISRCTN99248812, on 28 May 2020 (https://www.isrctn.com/ISRCTN99248812).

### Qualitative analysis

To analyse the training group’s responses to open-ended questions, we used a directed content-analysis approach [28], guided by the impact and outcomes theorised as relevant. The YPAG co-produced the initial framing framework and co-analysed the data. The framework was iterated and refined into a final version after discussion meetings. The scheme was validated by two independent coders and reliability calculated on 25% of the data, adopting a threshold of κ = 0.6 (substantial agreement [29]). Disagreements were resolved by discussion between original coders or adjudication by a third reviewer. The presence/absence of each code was recorded for each response. We separately analysed responses to questions related to: (a) impact of training, (b) use of peer support skills and (c) intentions to use skills. We also checked whether overarching categories emerged across the three sections.

## Results

### Participants

**Fig. 1:**
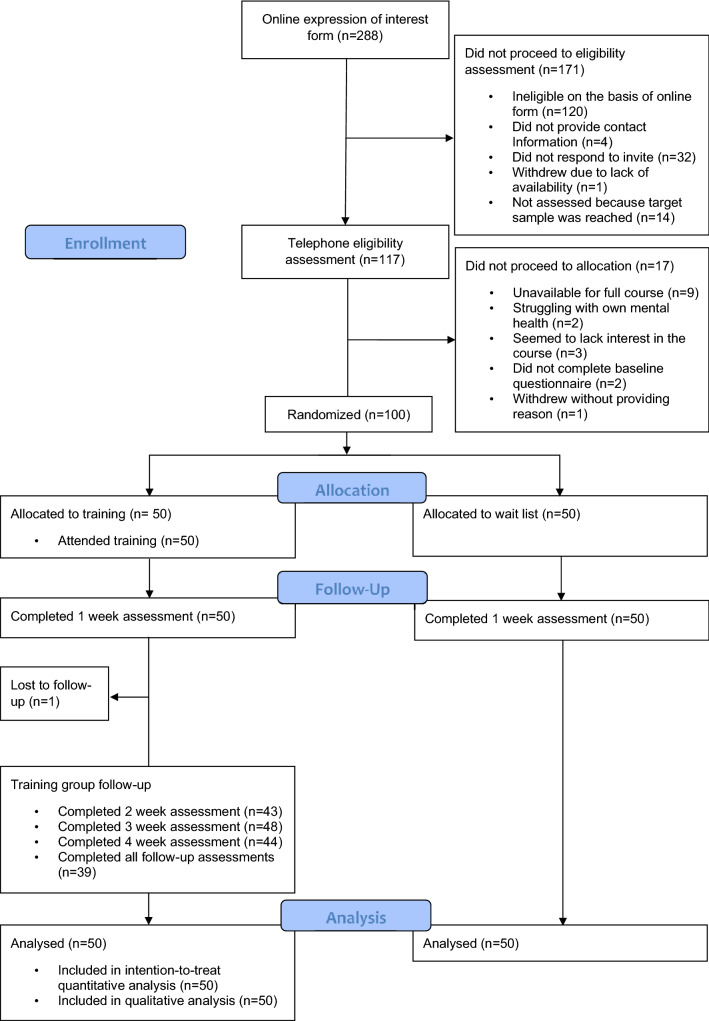
Progress of participants through the trial

We randomised 100 participants to either training (*n* = 50) or wait-list control (*n* = 50). Participants completed baseline assessments (30th May–3rd of June) and 1-week post-randomisation assessments (12–16th June 2020) during a lockdown period in the UK (with face-to-face teaching suspended from 20 March to 15 June 2020). Recruitment and retention rates are displayed in Fig. 1. All training group participants attended all training sessions, with the exception of one participant who missed one session. There were no dropouts in the 1-week assessment; however, one participant was lost to follow-up and others missed one or two follow-up questionnaires.

Baseline demographic characteristics are displayed in Table 1. Across both groups, most participants were aged 16 or 17 years (> 90%), self-identified as female (> 80%), and lived in England (> 80%). Both groups were ethnically diverse (< 50% White British), with similar levels of family affluence.

**Table 1:** Baseline demographic characteristics

	Training (*N* = 50)	Wait-list (*N* = 50)	Training vs Wait-list
*Age*			
16 years, *n* (%)	28 (56%)	37 (74%)	16 or 17 years: 18 years
17 years, *n* (%)	21 (42%)	10 (20%)	χ^2^ = 1.042, *p* = 0.307
18 years, *n* (%)	1 (2%)	3 (6%)	
*Gender identity* (*n,* %)			
Female	41 (82%)	43 (86%)	Female: Other
Male (cisgender or unspecified)	7 (14%)	7 (14%)	χ^2^ = 0.298, *p* = 0.585
Male (transgender)	1 (2%)		
Non-binary	1 (2%)		
*Family affluence scale*			*t* (98) = – 0.559, *p* = 0.577
Mean (SD)	6.22 (2.13)	6.44 (1.79)	
Low affluence (score 0–3), *n* (%)	7 (14%)	3 (6%)	
Medium affluence, (score 4–6) *n* (%)	16 (32%)	20 (40%)	
High affluence, (score 7–9) *n* (%)	27 (54%)	27 (54%)	
*Ethnicity* (*n*, %)			
White British	21 (42%)	24 (48%)	White British: Other
White Irish/White Other	9 (18%)	9 (18%)	χ^2^ = 0.364, *p* = 0.546
Black/Black British	7 (14%)	2 (4%)	
Mixed	1 (2%)	9 (18%)	
Asian/Asian British	9 (18%)	4 (8%)	
Chinese	1 (2%)	1 (2%)	
Other Ethnic group	2 (4%)	1 (2%)	
*Location*			England: Other
England	43 (86%)	40 (80%)	χ^2^ = 0.638, *p* = 0.424
Scotland	3 (6%)	3 (6%)	
Wales	2 (4%)	4 (8%)	
Northern Ireland	2 (4%)	3 (6%)	

### Quantitative results

Results from the ANCOVAs comparing training and wait-list groups on each primary and secondary outcome 1 week post randomisation, controlling for corresponding baseline score, gender and age are detailed in Table 2.

**Table 2:** Baseline and 1-week post-randomisation outcomes for training and wait-list control groups

Outcome	Training *N* = 50	Wait-list *N* = 50	ANCOVA*
Primary outcomes			
Ability to provide support	Mean (SD)	Mean (SD)	
*Motivation (ASCCD-COVID)*			*F*_1,95_ = 2.60, *p* = 0.110, η_p_^2^ = 0.03
Baseline	20.28 (3.05)	20.20 (3.31)	
Post	21.14 (2.76)	20.26 (3.22)	
*Skills (ASCCD-COVID)*			*F*_1,95_ = 15.83, *p* < 0.0001, η_p_^2^ = 0.14
Baseline	19.82 (4.21)	20.06 (4.38)	
Post	22.36 (3.49)	19.62 (4.93)	
*Frequency (ASCCD-COVID)*			*F*_1,95_ = 13.993, *p* < 0.0001, η_p_^2^ = 0.13
Baseline	15.92 (4.06)	17.04 (4.06)	
Post	19.66 (4.96)	17.46 (4.15)	
*Compassion to others-engagement*			*F*_1,95_ = 12.03, *p* = 0.001, η_p_^2^ = 0.11
Baseline	48.34 (6.58)	49.60 (7.17)	
Post	51.42 (6.11)	48.88 (7.28)	
*Compassion to others-Action*			*F*_1,95_ = 23.21, *p* < 0.0001, η_p_^2^ = 0.20
Baseline	31.24 (5.16)	31.94 (4.71)	
Post	34.28 (3.93)	31.32 (5.16)	
*Connectedness to peers*			*F*_1,95_ = 19.48, p < 0.0001, η_p_^2^ = 0.17
Baseline item score	4.18 (1.14)	4.08 (1.28)	
Post item score	5.34 (1.32)	4.08 (1.47)	
Secondary outcomes			
Mental wellbeing and emotional symptoms			
*Mental wellbeing (WEMWS)*			*F*_1,95_ = 62.51,
Baseline	47.48 (6.68)	46.84 (6.22)	*p* < 0.0001, η_p_^2^ = 0.40
Post	55.56 (6.30)	45.04 (9.28)	
*Emotional symptoms (SDQ-E)*			*F*_1,95_ = 8.26, *p* = 0.005, η_p_^2^ = 0.08
Baseline	4.16 (2.41)	4.62 (2.56)	
Post	2.84 (2.50)	4.36 (3.06)	
Agency (self-efficacy and civic engagement)			
*Self-efficacy (GSES)*			*F*_1,95_ = 7.91, *p* = 0.006, η_p_^2^ = 0.08
Baseline	30.92 (3.91)	31.12 (3.21)	
Post	33.16 (4.33)	31.32 (4.50)	
*Civic engagement-attitudes (CES-A)*			*F*_1,95_ = 25.82, *p* < 0.0001, η_p_^2^ = 0.21
Baseline	46.08 (5.61)	47.66 (6.52)	
Post	48.90 (5.62)	46.20 (7.39)	
*Civic engagement-behaviour (CES-B)*			*F*_1,95_ = 20.86, *p* < 0.0001,η_p_^2^ = 0.18
Baseline	30.80 (5.85)	30.38 (7.17)	
Post	34.34 (6.40)	28.44 (7.74)	

*Note.* η_p_^2^ = partial eta-squared. *ASCCD-COVID* Adolescent Social Connection Coping During COVID Scale. *WEMWS* Warwick–Edinburgh Mental Wellbeing Scale. *SDQ-E* Strengths and Difficulties Questionnaire-Emotional symptoms subscale. *GSES* General Self-Efficacy Scale. *CES-A* Civic Engagement Scale-Attitudes subscale. *CES-B*  Civic Engagement Scale-Behaviour subscale

*ANCOVA Analysis of Covariance, controlling for corresponding baseline score, gender (female versus all other genders) and age (age 16 versus age 17–18)

### Primary outcomes

#### Ability to provide support

There was no difference between the training and the wait-list groups on motivation to provide support to others (*F*_1,95_ = 2.60, *p* = 0.110). However, there was a significant effect of training on perceived support-giving skills (*F*_1,95_ = 15.83, *p* < 0.0001, η_p_^2^ = 0.14) and how often participants provided support to others (*F*_1,95_ = 13.99, *p* < 0.0001, η_p_^2^ = 0.13). Self-reported compassion towards others was also significantly greater among those who received training, compared to wait-list controls, both in relation to engaging with others and perceived ability to take action (*F*_1,95_ = 12.03, *p* = 0.001, η_p_^2^ = 0.11 and *F*_1,95_ = 23.21, *p* < 0.0001, η_p_^2^ = 0.20, respectively). The training group also reported feeling significantly more connected to their peers than wait-list controls (*F*_1,95_ = 19.48, p < 0.0001, η_p_^2^ = 0.17). With the exception of motivation, effect sizes across indicators of ability to provide support to others ranged from medium to large.

### Secondary outcomes

#### Mental wellbeing and emotional symptoms

We also found evidence of an effect of training on self-reported mental wellbeing and emotional symptoms. Those who received training reported significantly better mental wellbeing and significantly lower negative emotional symptoms compared to the wait-list, with large- and medium-effect sizes, respectively (*F*_1,95_ = 62.51, *p* < 0.0001, η_p_^2^ = 0.40, *F*_1,95_ = 8.26, *p* = 0.005, η_p_^2^ = 0.08).

#### Agency (self-efficacy and civic engagement)

Relative to the wait-list group, the training group reported significantly greater self-efficacy, with a medium-effect size (*F*_1,95_ = 7.91, *p* = 0.006, η_p_^2^ = 0.08). Compared to wait-list controls, the training group also reported more positive civic attitudes and greater perceived ability to engage in civic behaviours, each with a large-effect size (*F*_1,95_ = 25.82, *p* < 0.0001, η_p_^2^ = 0.21 and *F*_1,95_ = 20.86, *p* < 0.0001, η_p_^2^ = 0.18, respectively).

#### Training group follow-up

**Fig. 2:**
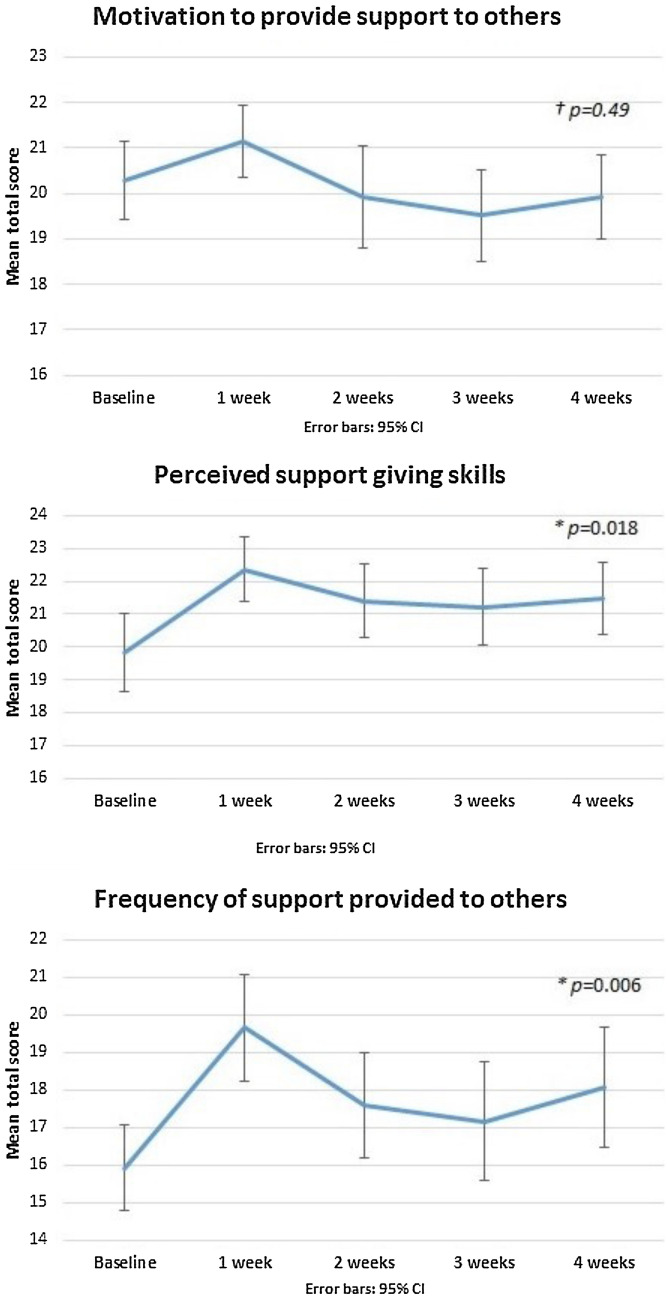
Indicators of ability to provide support among the training group from baseline to 4 weeks post randomisation; ^†^no significant difference between baseline and 4 weeks; *significant difference between baseline and 4 weeks

Participants most frequently reported helping close others: all training participants reported helping *friends* at least once over the follow-up period; 88% (*n* = 44) helped family members; 80% (*n* = 40) helped *other peers* and 38% (*n* = 19) helped *young people they did not know*. Figure 2 displays self-reported motivation to provide support to others, perceived support-giving skills and how often participants provided support to others at each assessment point among the training group. Repeated measures ANOVAs indicated perceived support-giving skills and how often participants provided support differed significantly across assessment points (*F*_2.65, 129.88_ = 6.13, *p* = 0.001; *F*_2.82, 138.08_ = 10.39, *p* < 0.001), and both increased from baseline to 4 weeks post randomisation (*p* = 0.018 and *p* = 0.006, respectively), although motivation at 4 weeks did not significantly differ from baseline (*p* = 0.49). The training group’s connectedness to their peers also differed across assessments (*F*_4, 196_ = 8.41, *p* < 0.0001), and participants reported feeling significantly more connected to their peers at 4 weeks compared to baseline (*p* = 0.001).

### Qualitative results

**Fig. 3:**
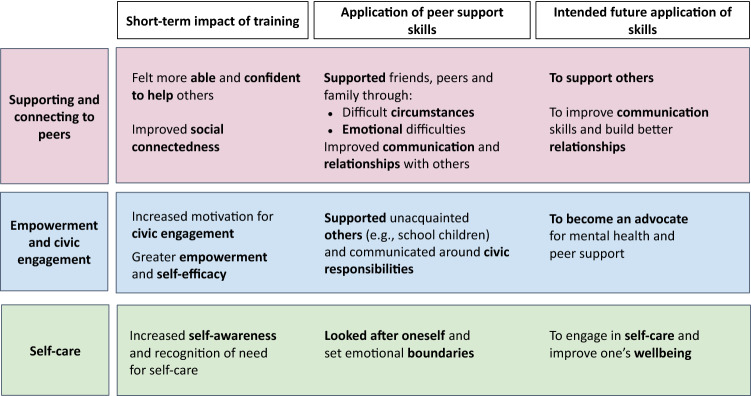
Impact of training, application of peer support skills and intended future use; content analysis main codes

Across the three open-ended questions, three main clusters emerged from participants’ responses: supporting and connecting with peers, empowerment and civic engagement, and self-care. Figure 3 provides a summary of the results; additional quotes and frequencies are provided in Table S3.

### Perceived impact of training

Increased ability to support and connect with peers was the main impact of the training. In particular, three-quarters of participants indicated that the training increased their ability and confidence in supporting others. For instance, Holly (all names are pseudonyms) expressed that the training made her “feel more prepared to help my friends and peers when they need me.” Two-fifths indicated that the training helped them build stronger relationships. For Paige, the training made her more “present within my friendships and relationships.”

About a third mentioned that the training made them feel empowered and more confident in themselves (“it made me see that I am more powerful and I can really make a change”; Rebecca), and had renewed aspirations to help the community (“I'm hoping to help my community increase the number of diverse Peer Supporters”, Charlotte). Equally frequent were references to increased self-awareness and self-care (“It has made me … more aware and more capable to deal with my own emotions and problems”; Stephanie).

Negative impacts of the training were rarely mentioned; only three participants indicated the training caused some fatigue (“made me tired due to lots of socialising and concentrating”; Kirsty), but no further negative experiences or harms were reported.

### Use of peer support skills

All participants reported at least one instance of using the skills to emotionally support others. Many young people described situational challenges peers faced, including lockdown, exam stress and family conflict (e.g., “I helped a friend who was struggling with being isolated in COVID-19 lockdown and just had a few conversations with them”; Georgia). Participants also reported specific emotional difficulties among their peers, and often mentioned techniques used to help others cope. Laura, for instance, supported a friend who “was having a panic attack and I tried to help her to calm down by suggesting that she did things which would distract her, I was referring to the emergency action plan which is something I learnt on the course.” Lastly, participants described using active listening skills; Jade, for example reported that “when having meaningful conversations I remembered to use a gentle tone and the mirroring technique.”

Two-fifths mentioned how their skills had been useful in helping the community and communicating around civic responsibilities. For example, Chloe reported that she used the skills “to help to educate others about the importance of understanding and fighting for the rights of those less privileged.” In addition, about a third mentioned using the skills for self-care and setting personal boundaries. Anna reported that “rather than shooting into panic mode, I reasoned with myself to think logically rather than emotionally, which had helped me calm down”.

### Intention to use the skills

All but one participant reported planning to use their new skills in the future, both immediately after the course (100%) and at the last follow-up (98%). The vast majority described plans to use the skills to support others (e.g., “I will try and help more of my peers even after all of this COVID-19 stuff has died down”; Georgina). Roughly half mentioned aspirations to improve their relationships and communication skills (e.g., “I plan to use some of the techniques like mirroring to help build stronger rapport with people”; Paul).

Many participants also reported a motivation to advocate for mental health and plans to contribute to community wellbeing. Paige reported she hoped to “set up a student organisation in my school, aiming to empower and educate younger students — with a focus on battling misogyny and sexual assault.” Participants also planned to continue to focus on self-care; for example, Samantha planned to “balance what I want to do and my own mental health.” Finally, an additional code identified in this section related to academic or professional aspirations, mentioned by about a fifth of participants. Alexandra, for instance, reported that “I hope to be a doctor so these skills will be extremely useful.”

## Discussion

This RCT documents benefits of an online peer support training model for young people during the COVID-19 pandemic across a range of outcomes. Although both the training and control participants expressed high motivation to support their peers, those who received training felt more able and likely to do so. These gains were maintained in the training group 3 weeks following course completion. The training group also felt more connected to other young people their age than controls. They most commonly reported using their skills to support friends with emotional difficulties and help them feel heard and appreciated. Critically, the training also improved participants’ own self-reported emotional symptoms and mental wellbeing, self-efficacy and civic engagement, relative to controls. Free text responses mirrored these findings, with qualitative analysis indicating that the training fostered support-giving skills, self-care, feelings of empowerment and a desire to help the community. Encouragingly, 3 weeks after training, participants continued to express intentions to apply the learned skills, not only to support themselves and others, but also to advocate for better mental health.

These results speak to the potential value of structured, targeted courses to train young people to provide peer support. While most peer support training courses are delivered face-to-face, our findings provide preliminary evidence of the potential benefits of delivering such courses online, leveraging platforms that are familiar to young people. This offers the potential to increase reach, and allows safe delivery during crises such as the COVID-19 pandemic [16]. Equipping young people with support-giving skills seemed to enable them to translate their motivation to help others into action. Although we did not provide youth with a structured opportunity to support others after the course, participants reported spontaneously offering help to their peers and family members, and felt more confident in doing so. Some indicated aspirations to pioneer peer support initiatives (e.g., discussion groups in schools) as a result of this training. It is possible that this opportunity to focus pro-socially on others, and to actively contribute to the pandemic response, alleviated feelings of uncertainty and honed young people’s sense of purpose during the pandemic, which may have contributed to the observed improvements in wellbeing [30, 31]. Our qualitative results also indicated that peer support training encouraged participants to practise self-care tactics in their daily life, which possibly also improved their wellbeing [32, 33].

## Limitations and future directions

To ascertain the short-term benefits of the course before a larger trial, our sample size was limited, and only immediate outcomes were assessed against a control group. Short-term follow-up assessments were evaluated in the training group only; therefore, it is difficult to determine whether the changes observed are due to the peer support training or other external factors. Future research should include a larger sample, and investigate potential medium- to long-term benefits of peer support training compared to controls. It would also be important to examine potential benefits for the supported peers and wider community. This is particularly relevant in the context of restrictions easing and young people returning to school and other social environments.

Our sample presented some diversity in ethnicity and socioeconomic status but lacked male representation. In addition, our sample was biased towards individuals who were already highly motivated to help others, which may have influenced their commitment to the course and positive outcomes. Approaches to widen participation co-directed through youth involvement will be important in future studies, and help ascertain whether gains are similar when training is delivered universally (e.g., through schools or primary care). Similarly, our sample size was not sufficient to explore outcomes among different subgroups of young people, for example outcomes among young people with high versus low wellbeing at baseline, and this will be an important consideration for future trials. Future research should also aim to compare the effects of peer support training and active controls. Similarly, is it also important to identify the mechanisms through which peer support training improves wellbeing, self-efficacy and civic engagement in young people, and what aspects of the course best predict these outcomes. A more comprehensive understanding of what works can help target training courses to particular populations and outcomes.

## Lessons learned and consequences for the future

Whilst the principle of youth involvement in designing research and interventions is increasingly accepted, young people's participation in priority setting is still limited. In this project we worked with groups of young people to identify how they wished to contribute to the pandemic response and co-created an online peer support training programme to facilitate this contribution. The active involvement of young people in setting the research agenda, and throughout the project, was key to meeting their aspirations and addressing their specific needs. Peer support training led to improvements in young people’s ability to help others, with additional benefits to their mental health and wellbeing and dimensions of agency (self-efficacy and civic engagement). These encouraging results suggest that online peer support training is a valuable tool to promote emotional support-giving skills in emergency situations such as COVID-19, paving the way for effective peer support interventions for youth wellbeing. Our research process, including mapping aspirations and supporting adolescents to take an active role in responding to a health crisis, provides a valuable model for co-creating effective research and interventions during times of instability and uncertainty.    

## Supplementary Information

Below is the link to the electronic supplementary material.

Supplementary file1 (PDF 255 KB)

## Data Availability

The quantitative dataset is available at https://osf.io/c2tk3/. Qualitative data are available upon reasonable request by emailing the corresponding author.

## Code availability (software application or custom code)

Available upon request.
