# Supplementary material for: Online peer support training to promote adolescents’ emotional support skills, mental health and agency during COVID-19: Randomised controlled trial and qualitative evaluation
Source: Eur Child Adolesc Psychiatry. 2022 Feb 17;32(6):1119–30. doi: 10.1007/s00787-021-01933-0 (PMC8853257; doi:10.1007/s00787-021-01933-0)
Supplement: Supplementary file 1 — Supplementary file1 (PDF 255 KB) [file 787_2021_1933_MOESM1_ESM.pdf]

Supporting Information for

Online peer support training to promote adolescents' emotional support skills, mental health and agency during COVID-19: Randomised controlled trial and qualitative evaluation

Gabriela Pavarini+, Tessa Reardon, Anja Hollowell, Vanessa Bennett, Emma Lawrance, Peer Support Young People's Advisory Group*, Vanessa Pinfold, Ilina Singh

*Ellie Brooks-Hall, Ashley Foster-Estwick, Damian Juma, Peter Lewis, Lucy Power, Maia Rogers

+Corresponding author: gabriela.pavarini@psych.ox.ac.uk

This file includes:

Tables S1 to S3

TableS1. Peer Support Young People's Advisory Group's contributions to the project

Number of meetings	Contribution
2 meetings and an online survey	Oxford researchers, the McPin Foundation and Senior NeurOX Young People's Advisory Group members co-led consultation with NeurOX YPAG members, the McPin Young People's Network and the Lancet Young Leaders for Global Mental Health to set research priorities. Peer Support YPAG was then formed comprising members of these three groups.
1	YPAG informed development of the outcome measures: they approved and adjusted quantitative scales and collaborated on the writing of qualitative questions.
2	YPAG consulted directly with the peer support training providers to advise on the content and structure of the course. For example, the YPAG emphasised the importance of teaching self-care, and of adapting the content to be culturally relevant for a UK audience (the training providers were USA based).
1	YPAG fed into dissemination plans to engage young people with the idea of peer support during Covid-19, including development of a social media campaign.
2	YPAG co-analysed the qualitative data, with training and support provided by the research team staff. Coding frameworks for each of the three qualitative questions were produced in the first of these meetings. For each of the three questions, initial codes were generated by one researcher and two YPAG members, such that all YPAG members contributed to the codes. These sets of codes were then iterated and refined into a final version after discussion meetings with the YPAG and research team. Coding was carried out in the second meeting and outside of meetings.

TableS2. Minor amendments to Compassion to Others-Action Subscale and Civic Engagement Scale-Behaviour Subscale

**Compassion to Others – Action Subscale**	
Original item	***Adapted item***
I direct attention to what is likely to be helpful to others.	I am able to direct attention to what is likely to be helpful to others.
I think about and come up with helpful ways for them to cope with their distress	I am able to think about and come up with helpful ways for them to cope with their distress
I take actions and do the things that will be helpful to others was changed to	I am able to take the actions and do the things that will be helpful to others
I express feelings of support, helpfulness and encouragement to others	I am able to express feelings of support, helpfulness and encouragement to others
**Civic Engagement Scale – Behaviour Subscale**	
Original item	***Adapted item***
I am involved in structured volunteer positions in my community	I am able to volunteer in my community
When working with others, I make positive changes in the community	When working with others, I can make positive changes in the community
I help members of my community	I can help members of my community
I stay informed of events in my community	I feel able to stay informed of events in my community
I participate in discussions that are relevant to the community	I feel able to participate in discussions that are relevant to the community
I contribute to projects to help the community	I feel able to contribute to projects to help the community

TableS3. Example quotes and frequencies of codes from content analysis (Note: reported percentages refer to proportion of participants whose answer(s) included the respective content; we only report codes that exceeded the threshold of 10%).

SHORT-TERM IMPACT OF TRAINING

**Overarching category**	**Code (%)**	**Example quotes**
**Supporting and connecting to peers**	Felt more able and confident to help others (74%)	“The training has helped me understand how I can better support my friends in the future.” [Laura, 16 years old]<br>“The training has taught me how to listen.” [Sarah, 16 years old]<br>“helped me to be able to talk and communicate with my friends and others.” [Katie, 16 years old]
	Improved social connectedness (40%)	“I feel I have a closer relationship to one of my friends who I have used my knowledge from the course to help.” [Lauren, 16 years old]<br>“I feel like I am part of something.” [Natalie, 17 years old]<br>“[helped] me build trusting new relationships and strengthen existing bonds.” [Anna, 16 years old]
**Empowerment and civic engagement**	Higher motivation for civic engagement (16%)	“[I] am much more motivated to make a change within my community.” [Georgina, 16 years old]<br>“I definitely feel more equipped to help others and make a change in my community” [Jennifer, age 16 years old]<br>“I'm much more aware of how our community needs people with Peer Supporter qualities.” [Charlotte, 16 years old]
	Greater empowerment and self-efficacy (34%)	“The training has allowed me to recognise my strengths and feel more confident in myself.” [Victoria, 17 years old]<br>“I have gained a lot more self esteem.” [Georgina, 16 years old]<br>“I think the training has definitely had a positive impact on how I see myself.” [Rachel, 16 years old]
**Self-care**	Increased self-awareness and recognition of need for self-care (32%)	“the training has also helped me try and understand myself better and embrace who I am (…) as well as the importance of self-care in my day-to-day life.” [Louise, 16]<br>“It has made me more aware of my self-care which in turn has helped my relationships with others. Now that I know what works for me in terms of self-care I feel I can utilise to not only help others but also calm me down in situations where I need a level head and concentration to help somebody”. [Eleanor, 17]<br>“I feel more confident and take more time in my day to use some of the self care tactics.” [Lauren, 16 years old]

**Hope**	Hopefulness and positivity for the future (12%)	“taught me the importance of being positive rather than focusing only on the negative and being pessimistic.” [Melissa, 17 years old]<br><br>“reinstated my sense of purpose- helping me feel more hopeful for the future.” [Paige, 17 years old]<br><br>“the course has helped me (…) be hopeful for the future.” [Paul, 17 years old]

USE OF PEER SUPPORT SKILLS

Overarching category	Code	Example quotes
**Supporting and connecting to peers**	Supported others through difficult circumstances (65.3%)	“I supported a friend who is having a hard time currently in lockdown due to living with a shielding family member.” [Chloe, 16 years old]<br><br>“I met up with a friend who has been struggling during lockdown and I think I used some of the skills from the course (…) to reframe their problems which is never done before and though was very useful.” [Rachel, 16 years old]<br><br>“One of my friends was upset over what career path to take and her sexuality, so I used the exercise where you reflect on her situation and say what strengths she has. I also used level 2 listening and didn’t give advice but just listened to her and show my support through that.” [Lauren, 16 years old]
	Supported others through emotional difficulties (69.4%)	“I helped my sister find self care tools to deal with her anxiety.” [Natalie, 17 years old]<br><br>“Helped my friends reframe their negative thoughts.” [Abigail, 17 years old]<br><br>“One of my close friends found themselves in a situation where they felt very negative about themselves (…). We analysed the situation and their response and said what was good about them through the actions they took. E.g they blocked the person who was harassing them and I said that by doing that was a demonstration of how strong they are.” [Lauren, 16 years old]
	Improved communication and relationships with others (71.4%)	“someone confided in me about them not feeling heard by the heads of the sixth form and I was there to listen to them and continuously ask myself "okay, why did they feel like this"? and be in touch with their emotions.” [Charlotte, 16 years old]<br><br>“What I found most useful is knowing that when someone wants to talk to me, they don't necessarily expect me to fix their problems, they just want me to listen. This was really important because it meant I could focus on just being there for them instead of worrying about trying to think of a solution.” [Amy, 16 years old]<br><br>“I have been using the rapport hacks in order to bond more effectively with my co-workers (…).” [Georgina, 16 years old]

**Empowerment and civic engagement**	Supported unacquainted others and communicated about civic responsibilities (38%)	“i offered a twelve year old girl support and space to express her feeling by showing that i relate to some of her troubles.” [Nicole, 17 years old]<br><br>“the rapport building skills were very helpful when reaching out to my friends and community.” [Peter, 17 years old]<br><br>“I was able to engage in a thoughtful discussion on issues I am passionate about that must be heard and taught about. They were difficult conversations to have on the crisis in Yemen and the Black Lives Matter Movement but they were respectful, necessary and even empowering in the sense that there was no more silence and stigma”. [Anna, 16 years old]
**Self-care**	Looked after oneself and set emotional boundaries (32.65%)	“I tried to stop myself from being over involved in their problems and trying to help them until I felt exhausted. Instead, I set boundaries and tried to stick to them.” [Louise, 16 years old]<br><br>“Since the course, my panic attacks have become less frequent (…) I think this is because I’m able to recognise escalation better and know what to do.” [Alex, 16]<br><br>“the coping skills taught in the training were very helpful when persevering through hard times.” [Peter, 17 years old]

INTENTIONS TO USE SKILLS

**Overarching category**	**Code**	**Example quotes**
**Supporting and connecting to peers**	To support others<br><br>(88% immediate outcome<br>61.4% follow-up week 4)	“I plan to use it when I help people who are under distress, as I feel more confident with dealing with those situations after the training.” [Danielle, 16 years old]<br><br>“I plan to use them myself to help support others when they are struggling”. [Louise, 16 years old]<br><br>"I plan to support those in my immediate community, such as people in my school or work." [Jennifer, 16 years old]
	To improve communication skills and build better relationships<br><br>(40% at immediate outcome<br>56.8% at follow up week 4)	“I’ll be able to understand the other person’s point of view better whilst not changing my personal beliefs which is a very important skill.” [Victoria, 17 years old]<br><br>"I plan to use the lessons I have learnt to better connect and communicate with people to provide support, show empathy and be able to comfort as well as empower them with my past experiences. I have learnt to use my pain to relate to others' suffering. The social skills I have picked up during the training in regards to the listening levels will allow me to be more engaged and focused when in conversation with others.” [Anna, 16 years old]

		"I want to change the way I approach conflict, and adjust the way I support my friends and family." [Natalie, 17 years old]
**Empowerment and civic engagement**	To become an advocate for mental health and peer support<br><br>(20% at immediate outcome<br>18.2% at follow-up week 4)	"I've already planned an assembly with the new year 7s to talk about boundaries in school and making the most of what it has to offer." [Alex, 16 years]<br><br>"I want to improve the peer monitoring in my school." [Samantha, 16 years old]<br><br>"I also hope to set up a form of wellbeing group at my new college so I can continue having such positive discussions with other young people." [Georgina, 16 years old]
**Self-care**	To engage in self-care and improve one's wellbeing<br><br>(18% at immediate outcome<br>25% at follow-up week 4)	"so I can balance what I want to do and my own mental health." [Samantha, 16 years old]<br><br>"learn how to take better care of myself." [Melissa, 17 years old]<br><br>"My skills will definitely prove very useful [...] especially since I'll need to be mindful of self-care and when to practise it." [Charlotte, 16 years old]
**Professional and academic aspirations**	To achieve professional and academic aspirations<br><br>(20% at immediate outcome<br>15.9% at follow-up week 4)	"I want to pursue a career as a safeguarding detective and I feel that this will provide me invaluable support." [Ricardo, 16 years old]<br><br>"I also hope the training will be useful in my future career as I want to go into medicine and feel the skills I learnt will benefit me in a lot of circumstances." [Eleanor, 17 years old]<br><br>"I am (hopefully) going into the field of law, and therefore certain subjects will greatly help me to be aware of the issues faced by individuals and how I can support those I may work with in human rights or criminal law." [Chloe, 16 years old]
